# Investigation of genetic polymorphisms associated with severe root resorption in orthodontic surgery patients

**DOI:** 10.1590/0103-644020266742

**Published:** 2026-06-26

**Authors:** Ângela Graciela Deliga Schröder, Milena Sampaio Kuczera, Cristiano Miranda de Araujo, Veridiana Pereira de Sá de Freitas, Michelle Nascimento Meger, Katheleen Miranda dos Santos, Rafaela Scariot, Erika Calvano Küchler, Flares Baratto-Filho

**Affiliations:** 1 Tuiuti University of Paraná, R. Padre Ladislau Kula, 395 - Santo Inácio, Curitiba, Brazil; 2 University of the Joinville Region, Rua Paulo Malschitzki, 10 - Zona Industrial Norte, Joinville, Santa Catarina, 89219-710, Brazil; 3 Department of Stomatology, School of Dentistry, Federal University of Paraná, Av. Prefeito Lothário Meissner, 632 - Jardim Botânico, Curitiba, PR, 80210-170, Brazil; 4 Department of Orthodontics, Medical Faculty, University Hospital Bonn, Welschnonnenstr. 17, 53111, Bonn, Germany

**Keywords:** Root resorption, genetics, genetic polymorphisms, orthodontic treatment, SMAD6, BMP2, RUNX2, BMP4

## Abstract

This study aimed to evaluate the association between genetic polymorphisms in the *BMP2, BMP4, RUNX2,* and *SMAD6* genes and the presence of severe external apical root resorption (EARR) in orthodontic patients undergoing orthognathic surgery. EARR represents a significant adverse outcome in orthodontic treatments, and genetic susceptibility may influence its occurrence. A cross-sectional study was conducted with 158 orthodontic patients treated with fixed appliances and mono or bimaxillary orthognathic surgery. Postoperative panoramic radiographs were used to assess severe EARR. Genomic DNA was extracted from buccal epithelial cells. Seven single-nucleotide polymorphisms (SNPs) in the target genes were genotyped using real-time PCR with TaqMan assays. Statistical analyses were performed with Chi-square or Fisher’s exact tests. No significant associations were observed for polymorphisms in *BMP2 (*rs1005464, rs235768)*, BMP4* (rs17563)*, RUNX2* (rs59983488, rs1200425)*,* or *SMAD6* (rs2119261*)*. However, a significant association was found for *SMAD6* rs3934908 **(**p < 0.05), with the CC genotype more frequent in patients with severe EARR. No deviations from the Hardy-Weinberg equilibrium were observed. The findings suggest that the *SMAD6 rs3934908* polymorphism may be associated with an increased susceptibility to severe EARR in orthodontic surgery patients. While most SNPs showed no association, these results contribute to understanding the genetic factors involved in EARR. Further studies with larger samples are needed to confirm these findings and explore gene-environment interactions.

## Resumo

Este estudo teve como objetivo avaliar a associação entre polimorfismos genéticos nos genes BMP2, BMP4, RUNX2 e SMAD6 e a presença de reabsorção radicular apical externa severa (EARR) em pacientes ortodônticos submetidos à cirurgia ortognática. A RRAE representa um importante desfecho adverso nos tratamentos ortodônticos, e a suscetibilidade genética pode influenciar sua ocorrência. Foi conduzido um estudo transversal com 158 pacientes ortodônticos tratados com aparelhos fixos e submetidos à cirurgia ortognática mono ou bimaxilar. Radiografias panorâmicas pós-operatórias foram utilizadas para avaliar a presença de RRAE severa. O DNA genômico foi extraído de células epiteliais bucais. Sete polimorfismos de nucleotídeo único (SNPs) nos genes-alvo foram genotipados por meio de PCR em tempo real utilizando ensaios TaqMan. As análises estatísticas foram realizadas utilizando os testes Qui-quadrado ou teste exato de Fisher. Não foram observadas associações significativas para os polimorfismos em BMP2 (rs1005464, rs235768), BMP4 (rs17563), RUNX2 (rs59983488, rs1200425) ou SMAD6 (rs2119261). No entanto, foi identificada uma associação significativa para o polimorfismo SMAD6 rs3934908 (p < 0,05), sendo o genótipo CC mais frequente em pacientes com EARR severa. Não foram observados desvios do equilíbrio de Hardy-Weinberg. Os achados sugerem que o polimorfismo SMAD6 rs3934908 pode estar associado a uma maior suscetibilidade à RRAE severa em pacientes submetidos à cirurgia ortognática. Embora a maioria dos SNPs avaliados não tenha demonstrado associação, esses resultados contribuem para a compreensão dos fatores genéticos envolvidos na RRAE. Estudos futuros com amostras maiores são necessários para confirmar esses achados e explorar possíveis interações gene-ambiente.

**Figure:**
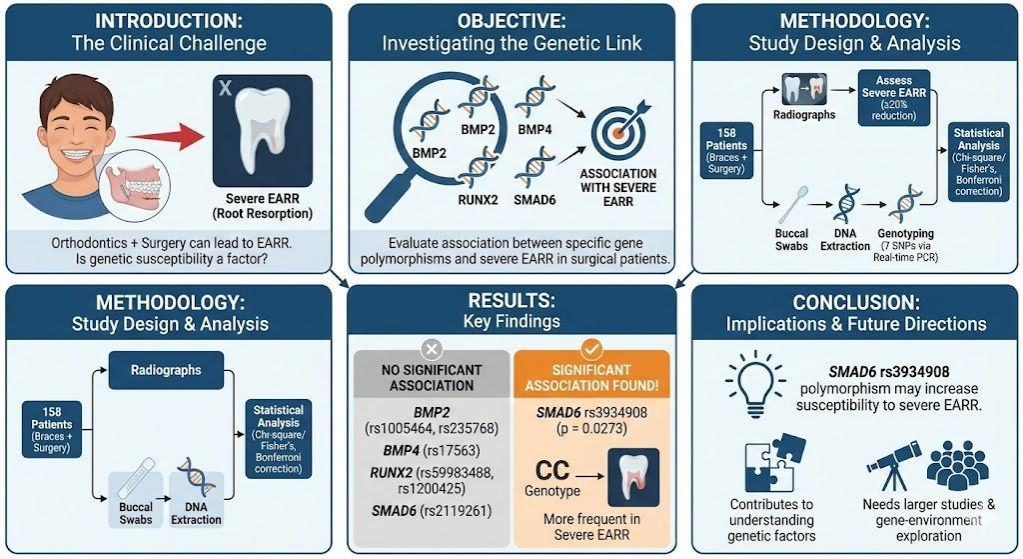

## Introduction

External apical root resorption (EARR) is a relatively common biological consequence of orthodontic tooth movement, characterized by the permanent loss of root structure from the apex^1^ ^,^ ^2^. While in most patients this phenomenon is minor and clinically insignificant, in rare cases it may become severe, potentially compromising the integrity of the affected teeth and limiting orthodontic outcomes^3^ ^,^ ^4^ ^,^ ^5^ ^,^ ^6^.

EARR occurs when the protective layer of cementoblasts covering the root surface is damaged or removed, exposing the root to osteoclastic activity and leading to resorption^4^. Although mechanical forces applied during orthodontic treatment are necessary for tooth movement, not all individuals experience EARR to the same extent. This has led to the longstanding hypothesis that individual susceptibility plays a critical role in the severity of resorption, independent of systemic or treatment-related variables^4^ ^,^ ^6^.

Efforts to identify predictors of EARR have pointed to a potential genetic component, as clinical observations suggest a familial or individual predisposition. However, the specific mechanisms underlying this susceptibility remain poorly understood, and no definitive genetic markers have yet been established^5^ ^,^ ^7^ ^,^ ^8^.

Bone morphogenetic proteins (*BMP*s), including *BMP2* and *BMP4*, are signaling molecules that belong to the transforming growth factor-beta (*TGF-β*) superfamily. They are involved in key processes related to craniofacial development and osteogenesis. Previous studies have reported associations between *BMP2* and *BMP4* gene polymorphisms and oral clefts or dental anomalies ^9^ ^,^ ^10^, suggesting a plausible role in root development and remodeling. Given their involvement in tooth morphogenesis and skeletal homeostasis, it is biologically plausible that variations in these genes may contribute to individual susceptibility to EARR.

Moreover, *SMAD6*, a negative regulator of the BMP/TGF-β signaling pathway, plays a crucial role in modulating osteogenic activity. Variants such as rs2119261 and rs3934908 may impair the inhibitory regulation of *SMAD1/5/8* signaling, potentially leading to exaggerated bone remodeling and, consequently, an increased risk of EARR in orthodontic patients. Therefore, we hypothesize that *SMAD6* polymorphisms contribute to individual susceptibility to EARR.

Importantly, several genes have already been investigated in relation to EARR, particularly those involved in inflammation and bone remodeling, such as *IL1A, IL1B, IL1RN, IL6, RANK, RANKL, OPG, P2RX7, IRAK1, SPP1,* and *VDR*. A systematic review by Pinheiro et al. (2021) summarized the available evidence and highlighted that multiple genetic variants have been associated with increased susceptibility to EARR, although the overall certainty of the evidence remains low^11^. Situating the present study within this context reinforces the need to investigate additional candidate genes with plausible biological roles, such as *RUNX2, BMPS,* and *SMAD6.*

Although EARR is clinically relevant^12^ and there is a growing interest in its genetic determinants, few studies have explored the association between candidate gene polymorphisms and the occurrence of severe root resorption in orthodontic patients. Furthermore, to date, no studies have been identified in the literature focusing specifically on orthodontic patients undergoing orthognathic surgery, who are subjected to particularly complex biomechanical conditions.

Therefore, this study aimed to investigate the possible association between genetic polymorphisms in *BMP2* (rs1005464 and rs235768), *BMP4* (rs17563), *RUNX2* (rs59983488 and rs1200425), and *SMAD6* (rs2119261 and rs3934908) and the occurrence of EARR in a sample of orthodontic patients who underwent orthognathic surgery. By addressing a specific gap in understanding the genetic predisposition to EARR, this study helps clarify the potential role of bone-related signaling pathways in the etiology of severe root resorption. The findings help inform risk assessment strategies and support the development of more personalized orthodontic care in the future.

## Materials and methods

### Study Design and Ethical Approval

This cross-sectional study was conducted in accordance with the STREGA (Strengthening the Reporting of Genetic Association Studies) guidelines^12^ and was approved by the Research Ethics Committee of the University Positivo local committee under protocol number: 69240817.7.0000.0093.

### Setting

**Figure 1:**
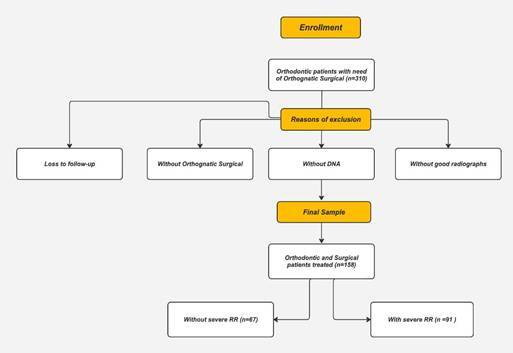
Flowchart of sample selection. From the initial 310 orthodontic patients with an indication for orthognathic surgery, exclusions were applied due to loss to follow-up, absence of orthognathic surgery, unavailable DNA, or inadequate radiographs. The final sample comprised 158 orthodontic and surgical patients, divided into those without severe root resorption (n=67) and those with severe root resorption (n=91)

Patients from both sexes who had monomaxillary or bimaxillary orthognathic surgery as part of their orthodontic treatment with fixed appliances at one University were included in the study. Orthodontic residents treated all patients under faculty supervision. For each of the 158 patients who met the inclusion criteria (Figure 1), data were recorded on age, sex, ethnicity, dental classification (based on Angle’s system), skeletal classification (based on Steiner’s analysis), and ANB angle. Each patient was also assessed for the presence of EARR.

### Eligibility Criteria

Eligibility criteria were as follows: patients aged 18 years or older; undergoing orthodontic treatment with a formal indication for orthognathic surgery; availability of high-quality panoramic radiographs taken both preoperatively and immediately postoperatively; no history of systemic diseases affecting bone metabolism; and no prior orthodontic treatment before surgical preparation.

Exclusion criteria included a history of craniofacial syndromes or congenital craniofacial anomalies, previous dentoalveolar trauma, endodontic treatments, open root apices or incomplete root development, and incomplete or technically compromised radiographic documentation.

With regard to potential confounding factors, our study included only patients over 18 years of age, of both sexes, thereby reducing heterogeneity related to dental and skeletal maturity. The duration of orthodontic treatment is universally recognized as a relevant biomechanical risk factor and a potential confounder; therefore, in the present study, all patients underwent orthodontic treatment for approximately 24-30 months prior to surgery. In addition, the surgical procedures encompassed maxillary, mandibular, or combined osteotomies. Controlling for these elements contributes to methodological rigor and strengthens the internal validity of the findings, minimizing potential biases that could affect the association between the investigated genetic polymorphisms and the occurrence of EARR.

### Radiographic Evaluation

Panoramic radiographs obtained immediately after orthognathic surgery were analyzed by a dental radiologist with expertise in oral and maxillofacial radiology. Each image was classified as showing the presence or absence of severe root resorption. All teeth were evaluated and the root resorption was assessed according to the classification proposed by Levander and Malmgren (1988) ^13^, which categorizes resorption into five distinct levels: Grade 0 (no visible signs of resorption); Grade 1 (irregular root apex contour); Grade 2 (resorption less than 2 mm of the root length); Grade 3 (resorption between 2 mm and one-third of the root length); and Grade 4 (severe resorption exceeding one-third of the root length). Only EARR defined as Grade 4, in at least one tooth, was included in this study.

To assess intra-examiner reliability in identifying the presence of severe root resorption, a set of 10 randomly selected radiographs was reanalyzed by the same examiner after a two-week interval. Agreement between evaluations was assessed using the Kappa coefficient, based on the presence or absence of severe resorption. According to the classification proposed by Landis and Koch^14^, Kappa values greater than 0.80 were interpreted as indicating excellent agreement.

### Genotyping

Genomic DNA was isolated from oral epithelial cells collected via saliva, following a previously established protocol^10^. The concentration and purity of the extracted DNA were assessed using spectrophotometry (NanoDrop 1000; Thermo Scientific). For this study, seven single-nucleotide polymorphisms (SNPs) from four genes involved in bone remodeling and craniofacial development (*BMP2*, *BMP4*, *RUNX2*, and *SMAD6*) were selected, based on prior evidence of their association with dental anomalies and skeletal traits, as well as their biological roles in tooth and bone development (see Table 1). Genotyping was performed in a blinded manner using real-time polymerase chain reaction (PCR) with TaqMan assays (Step One Plus Real-Time PCR System; Applied Biosystems). Each reaction was performed in a final volume of 5 μL, containing 2.5 μL of TaqMan Genotyping Master Mix, 0.125 μL of the specific SNP assay (Applied Biosystems), and four ng of genomic DNA diluted in water. Thermal cycling conditions included an initial denaturation at 95 °C for 10 minutes, followed by 40 cycles of amplification at 92 °C for 15 seconds and 60 °C for 1 minute. Detailed information regarding the selected polymorphisms is provided in Table 2 (MISSING).

Sample size estimation was performed using G*Power software (version 3.1.9.7), based on a Chi-square test for the association between genotype and the dichotomous outcome of severe EARR. Assuming a 30% prevalence of EARR in the risk genotype group and 10% in the reference group, with α = 0.05 and 80% power, the minimum sample size required was estimated to be 144 individuals (44 72 per group). These parameters were chosen based on typical effect sizes reported in genetic association studies of dental traits, following Cohen’s recommendations for moderate effects^15^.

### Statistical Analysis

Statistical analyses were performed using GraphPad Prism version 9 (GraphPad Software). The frequency of severe root resorption among different genotype groups was compared using the Chi-square test.

**Table 1:** Genotype frequencies of candidate gene polymorphisms in patients with and without severe external apical root resorption (EARR)

Gene/SNP	Genotype	Without EARR (n, %)	With EARR (n, %)	P-value
BMP2 (rs1005464)	GG	44 (65.67%)	60 (65.93%)	0,9919
	GA	19 (28.36%)	26 (28.57%)	
	AA	4 (5.97%)	5 (5.49%)	
BMP2 (rs235768)	TT	28 (41.79%)	41 (46.07%)	0,5093
	TA	29 (43.28%)	40 (44.94%)	
	AA	10 (14.93%)	8 (8.99%)	
BMP4 (rs17563)	AA	20 (29.41%)	29 (31.87%)	0,6884
	AG	38 (55.88%)	45 (49.45%)	
	GG	10 (14.71%)	17 (18.68%)	
RUNX2 (rs59983488)	GG	36 (53.73%)	59 (68.60%)	0,1314
	GT	30 (44.78%)	25 (29.07%)	
	TT	1 (1.49%)	2 (2.33%)	
RUNX2 (rs1200425)	AA	13 (19.12%)	20 (22.47%)	0,0823
	AG	37 (54.41%)	33 (37.08%)	
	GG	18 (26.47%)	36 (40.45%)	
SMAD6 (rs2119261)	CC	21 (30.88%)	37 (42.05%)	0,2325
	CT	31 (45.59%)	38 (43.18%)	
	TT	16 (23.53%)	13 (14.77%)	
SMAD6 (rs3934908)	CC	7 (10.61%)	25 (27.78%)	0,0273
	CT	39 (59.09%)	46 (51.11%)	
	TT	20 (30.30%)	19 (21.11%)	

## Results

**Figure 2:**
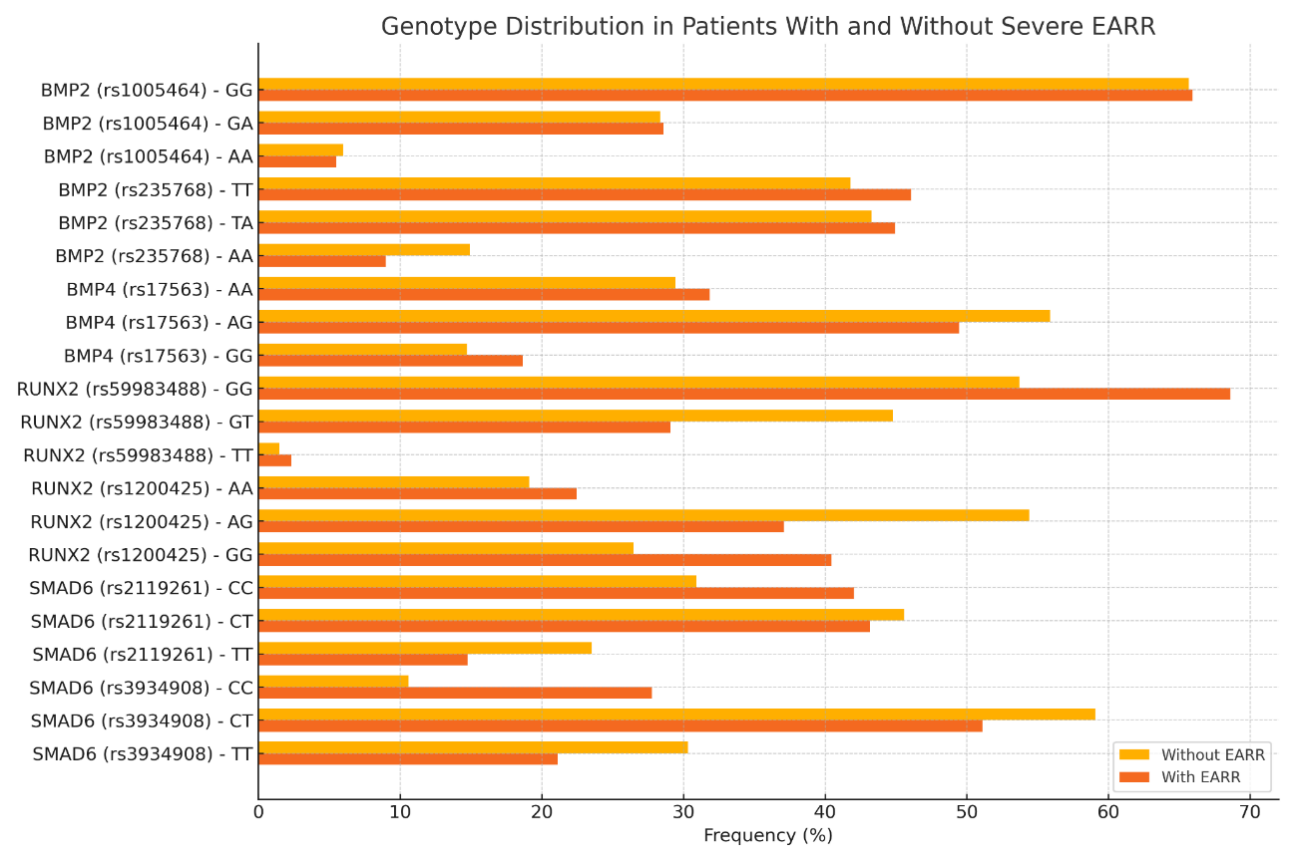
Comparison of genotype frequencies for *BMP2* (rs1005464, rs235768), *BMP4* (rs17563), *RUNX2* (rs59983488, rs1200425), and *SMAD6* (rs2119261, rs3934908) between orthodontic patients with and without severe external apical root resorption (EARR).

A total of 158 orthodontic surgery patients were evaluated for the presence or absence of severe EARR. Genotype frequencies of selected polymorphisms in *BMP2*, *BMP4*, *RUNX2*, and *SMAD6* genes were compared between individuals with and without severe EARR (Figure 2). The distribution of genotypes and corresponding statistical analyses are presented in Table I.

For *BMP2* rs1005464, genotype frequencies were similar between patients without EARR (GG: 65.67%, GA: 28.36%, AA: 5.97%) and those with severe EARR (GG: 65.93%, GA: 28.57%, AA: 5.49%), with no statistically significant difference observed (*p* = 0.9919). Similarly, for *BMP2* rs235768, the distribution of TT, TA, and AA genotypes showed no significant difference between groups (*p* = 0.5093).

In the case of *BMP4* rs17563, genotype frequencies (AA, AG, GG) did not significantly differ between the non-resorption and resorption groups (*p* = 0.6884).

For *RUNX2* rs59983488, although the GG genotype was more frequent in the EARR group (68.60%) compared to the non-EARR group (53.73%), and the GT genotype was correspondingly less frequent (29.07% vs. 44.78%), this difference did not reach statistical significance (*p* = 0.1314). Similarly, *RUNX2* rs1200425 did not show a statistically significant association with severe EARR, despite an apparent higher frequency of the GG genotype in the resorption group (40.45% vs. 26.47%; *p* = 0.0823).

For *SMAD6* rs2119261, genotype distribution also did not significantly differ between groups (*p* = 0.2325). However, for *SMAD6* rs3934908, a significant difference in genotype distribution was observed between groups (*p* = 0.0273). The CC genotype was more frequent in patients with severe EARR (27.78%) compared to those without (10.61%), while the CT and TT genotypes were less frequent in the resorption group.

No deviations from the Hardy-Weinberg equilibrium were observed for any of the studied polymorphisms in either group (data not shown).

## Discussion

This study evaluated the association between seven polymorphisms in *BMP2*, *BMP4*, *RUNX2*, and *SMAD6* genes and the presence of severe external apical root resorption (EARR) in orthodontic patients undergoing orthognathic surgery. Although most single-nucleotide polymorphisms (SNPs) did not show statistically significant associations, the *SMAD6* rs3934908 variant demonstrated a notable correlation, with the CC genotype being more frequent among individuals affected by severe EARR. This result suggests a potential genetic susceptibility modulated by the *SMAD6* pathway, contributing to the multifactorial etiology of root resorption and rejecting the null hypothesis for this specific locus^10^ ^,^ ^16^ ^,^ ^17^.

The most significant finding of this study was the association between the *SMAD6* rs3934908 polymorphism and severe EARR. Individuals with the CC genotype were more likely to present root shortening ≥ 20%, indicating a potential genetic predisposition modulated by impaired *BMP* inhibition. *SMAD6* encodes an intracellular antagonist of *BMP* signaling, involved in regulating bone remodeling and inflammatory responses^10^. The biological plausibility of this result aligns with the role of *BMP* signaling in root integrity, supporting prior evidence of *BMP*-related genes in craniofacial development and bone homeostasis^11^. These findings reinforce the need for genetic screening strategies in patients at higher risk of severe EARR, particularly those undergoing orthognathic surgery^18^ ^,^ ^19^.

The selected sample consisted of patients undergoing mono- or bimaxillary orthognathic surgery, a subgroup exposed to complex mechanical forces and potential surgical trauma(18, 19). Clinical data indicate that this group has a higher incidence of EARR compared to non-surgical orthodontic patients, with some studies reporting up to 23.4% of significant root resorption in bimaxillary procedures^19^. These findings suggest that the mechanical environment in surgical orthodontics may interact with genetic predisposition, exacerbating resorptive responses. The study’s focus on this high-risk population adds value by targeting a clinically vulnerable group and may help in identifying individuals requiring additional monitoring or adjusted force protocols during treatment^20^ ^,^ ^21^.

The multi-faceted biomechanical and surgical demands of combined orthodontic-surgical therapy, essential for correcting dentofacial deformities, impose significant biological stress on the dental roots, potentially exacerbating the individual predisposition to EARR. This susceptibility is hypothesized to be modulated by genetic factors, such as *SMAD6*. An elevated expression or activity of *SMAD6* would act as a crucial negative regulator, inhibiting the Bone Morphogenetic Protein (BMP) signaling pathway responsible for cementum repair. Consequently, this genetic vulnerability makes the root more susceptible to the mechanical and inflammatory insults arising from orthodontic preparation and surgical trauma. Clinically, this genetic risk necessitates a personalized treatment approach. The identification of *SMAD6*-related polymorphisms could guide the orthodontist in adopting targeted preventive strategies, such as utilizing lighter, intermittent forces with planned resting periods, thereby minimizing EARR risk. This conscientious planning protects dental longevity and, critically, preserves the patient's Oral Health-Related Quality of Life (OHRQoL), ensuring that the considerable time and resource investment in orthognathic surgery is not undermined by the long-term aesthetic and functional consequences of severe root structure loss ^26^ ^,^ ^27^ ^,^ ^28^ ^,^ ^29^. 

Although the other analyzed SNPs did not show statistically significant associations after correction, some trends were observed-particularly with *RUNX2* rs59983488 and rs1200425. For instance, a higher frequency of the GG genotype of rs59983488 was noted among patients with EARR. *RUNX2* is a transcription factor essential for osteoblastic differentiation and skeletal morphogenesis, and prior research has suggested its involvement in susceptibility to root anomalies^10^ ^,^ ^11^. While the current sample may not have had the power to detect modest associations, these patterns suggest that further investigation in larger cohorts is warranted to clarify the potential contribution of these variants to root resorption.

The findings of this study align with the widely accepted view that EARR is a multifactorial condition influenced by both environmental and genetic factors^22^ ^,^ ^23^ ^,^ ^24^. Historical and contemporary literature support a genetic basis for individual susceptibility to root resorption^3^ ^,^ ^4^ ^,^ ^25^. Clinical observations consistently report differential resorptive outcomes among patients subjected to similar orthodontic forces, indicating the relevance of hereditary traits^3^ ^,^ ^23^. Moreover, polymorphisms in genes related to bone remodeling, such as *VDR* and *BMP*s, have previously been implicated in resorptive processes^16^ ^,^ ^17^, which reinforces the biological rationale for investigating these markers in orthodontic contexts.

In addition, the findings of this study have important clinical implications for orthodontic-surgical practice. Recognizing the genetic susceptibility to EARR may support risk stratification of patients, guiding the adoption of more individualized protocols. For those at higher risk, orthodontists may consider applying lighter forces, scheduling more frequent radiographic monitoring, and ensuring closer integration between orthodontic and surgical teams to minimize complications. These preventive measures not only preserve root structure and dental longevity but also positively impact oral health-related quality of life by preventing functional and aesthetic impairments that could compromise the overall success of treatment.

A limitation of this study lies in the relatively small size of some genotypic subgroups, especially for rare alleles, which may have reduced the statistical power to detect subtle effects. Nonetheless, the rigorous radiographic protocol, standardized genotyping procedures, and focus on a high-risk clinical population strengthen the validity of the findings. The study also reinforces the novelty of *SMAD6* as a candidate gene for EARR, contributing valuable insights to the literature on gene-environment interactions in orthodontics. Future research should include larger and more ethnically diverse populations and explore functional pathways to validate these associations. Such efforts may ultimately enable the incorporation of genetic markers into individualized risk prediction models, advancing personalized orthodontic care.

## Conclusion

This study identified a possible association between the *SMAD6 rs3934908* polymorphism and individual susceptibility to severe external apical root resorption in orthodontic patients undergoing orthognathic surgery. Although most of the analyzed *SNPs* did not show significant associations, these findings support the contribution of genetic factors to the etiology of root resorption. Further studies with larger cohorts and functional analyses are needed to confirm these associations and clarify the underlying mechanisms.

## Data Availability

The research data are available upon request.
